# Endovascular Management of Intracranial Atherosclerotic Stenosis: Lessons from Mistakes in the Past and how to Achieve Improved Outcomes

**DOI:** 10.1007/s00062-021-01005-5

**Published:** 2021-03-09

**Authors:** Hans Henkes, Pervinder Bhogal, Victoria Hellstern, Hansjörg Bäzner

**Affiliations:** 1 Neuroradiologische Klinik, Katharinenhospital, Klinikum Stuttgart, Kriegsbergstraße 60, 70194 Stuttgart, Germany; 2 Medical Faculty, University Duisburg-Essen, Essen, Germany; 3 Department of Interventional Neuroradiology, The Royal London Hospital, London, UK; 4 Neurologische Klinik, Katharinenhospital, Klinikum Stuttgart, Kriegsbergstraße 60, Stuttgart, 70194 Germany

## Why Dealing with Intracranial Atherosclerotic Stenosis?

The term intracranial atherosclerotic stenosis (ICAS) refers to the lumen loss or occlusion of an intracranial artery due to an atherosclerotic plaque. With the widespread use of transcranial Doppler ultrasonography (TCD), computed tomography angiography (CTA), and magnetic resonance angiography (MRA), the prevalence of ICAS has increased during the last 25 years. About 8–10% of ischemic strokes in Europeans are now related to ICAS, with significantly higher numbers in African Americans, Asians, and Hispanics. The occurrence of ICAS is associated with advanced age, diabetes mellitus, arterial hypertension, peripheral atherosclerosis, and coronary heart disease. An ICAS-induced stroke can be due to arterio-arterial emboli, thrombotic vessel occlusion, hemodynamic compromise, perforator occlusion, and any combination thereof [1].

The WASID trial compared high-dose aspirin and monitored warfarin to treat ≥50% large intracranial vessel atherosclerotic stenosis. During a mean follow-up of 1.8 years, the rate of ischemic stroke in an artery’s supply territory with atherosclerotic stenosis was 15% and 12%, respectively [2]. In the SAMMPRIS trial, the primary endpoint was any stroke or death within 30 days after enrolment, ischemic stroke in the qualifying artery territory beyond 30 days of enrolment, or any stroke or death within 30 days after a revascularization procedure. During a median follow-up of 32.4 months, a primary endpoint event occurred in 15% of the patients undergoing aspirin and clopidogrel medication plus management of vascular risk factors and lifestyle modification [3].

## Medicinal Treatment

Warfarin has been shown to carry higher hemorrhagic risks than antiplatelet medication without added protective value against ischemic events [2]. The Platelet-Oriented Inhibition in New TIA and Minor Ischemic Stroke (POINT) trial compared aspirin alone versus clopidogrel plus aspirin for the secondary prevention after minor stroke or transient ischemic attack (TIA). The inclusion criteria did not address ICAS specifically. Clopidogrel plus aspirin and aspirin alone were associated with 5% and 6.5% major ischemic events, respectively. Major hemorrhage occurred in 0.9% and 0.4%, respectively [4].

In the SAMMPRIS trial, patients received 325 mg aspirin daily for the duration of the follow-up (and probably beyond) plus 75 mg clopidogrel daily for 90 days. This medication’s impact remains challenging to determine since the patients were also subject to lifestyle modification and vascular risk factor management [1]. Ticagrelor and prasugrel in secondary stroke prevention in patients with symptomatic ICAS are not yet well defined.

## Endovascular Treatment

Interventional cardiology techniques mainly influenced the underlying concept of treating high-grade ICAS by endovascular means. During the initial phase of this experience, dedicated balloon catheters for intracranial angioplasty were not yet available [5]. If well-selected patients were treated in experienced centers, high success rates and severe complication rates below 10% were achieved with balloon-expandable coronary stents [6, 7]. It became evident that the results in terms of periprocedural safety were good in elective cases, while the acute stroke setting was associated with a much higher complication rate [8]. The Pharos Vitesse stent (Micrus) was the first balloon-expandable stent with a dedicated indication for ICAS and was a derivative of a coronary stent. The initial results were quite promising [9]. In the VISSIT trial, patients were randomized for balloon-expandable stent treatment plus medical treatment or medical treatment alone. The stent group patients had a significantly higher rate of primary safety endpoint occurrence than the patients in the medical arm, including an 8.6% rate of intracranial hemorrhage [10]. A similar sequence of results had previously been observed for the Wingspan stent (Stryker). This is a combination of a non-compliant balloon and a self-expanding nitinol stent. The stent structure is the same as Neuroform, but the radial force is increased [11]. Again, the initial results were good, even though a completely new treatment technique had to be adopted [12]. In the SAMMPRIS trial medical treatment with aspirin and clopidogrel, vascular risk factor management and lifestyle modification were randomized against the same regimen plus balloon angioplasty and Wingspan implantation. In the Wingspan vs. the conservative management groups, any stroke rate was 26% vs. 19%, with hemorrhage rates of 13% vs. 4%, respectively [3]. Besides, follow-up examinations revealed a high rate of symptomatic in-stent stenosis of 14% at 3 years [13]. Concerns related to methodological aspects of SAMMPRIS have been published [14, 15]. On 15 September 2016, the Federal Joint Committee (Gemeinsamer Bundesausschuss) on behalf of the German Federal Ministry of Health ruled that the usage of stents for the treatment of intracranial stenosis is no longer covered by the health insurance companies concerned [16]. Exempted are patients with a ≥ 70% intracranial stenosis who had an infarct related to this stenosis and suffered from a second infarct despite intensive medical treatment. In addition, patients with an acute intracranial occlusion without a therapeutic alternative or after the failure of such an alternative are also included in the exemption.

## Future Directions

The current issue of *Clinical Neuroradiology* features three original articles dealing with the treatment of ICAS. Wang et al. used drug-coated balloons (DCB) without stenting in 35 patients and encountered significant complications in 2 (5.7%) patients [17]. In a similar study, Remonda et al. also used DCBs with only 2/33 (6%) intracranial complications without severe clinical sequelae. They reported, however, a 12% rate of symptomatic restenosis [18]. Guan et al. were able to demonstrate the spatial relationship between atherosclerotic plaques of the proximal middle cerebral artery and the origin of the lenticulostriate perforators [19]. These three papers illustrate the current attempts to improve the diagnosis and endovascular treatment of patients with ICAS.

There are several lessons learned from previous randomized trials and published papers.

*Conservative management *is a viable option for many patients and should be the first step, typically based on dual antiplatelet medication, monitored by adequate response tests (e.g., VerifyNow) to identify non-responders.

*Qualification of the interventionist* is a delicate subject. Annual minimum quantities of specific procedures per interventionist could be a means to avoid the risks associated with so-called low-volume centers. Active quality (i.e., outcome) management with external auditing would be a further step ahead.

*Timing* of the endovascular treatment is crucial, and procedures within the first day(s) after the index event are hazardous [20].

*Logistics* are of utmost importance. Patients with ICAS need continuous monitoring and aggressive blood pressure management after the removal of the intracranial stenosis. To some extent, systolic blood pressure below 120 mm Hg is a safeguard against reperfusion hemorrhage [21].

*The morphology* and anatomy of ICAS plaques are to a certain extent varied. It is unlikely that a single device or method can address different target lesion types. For many intradural stenoses (e.g., M1 segment), balloon angioplasty without a stent is a good option [22]. Drug-eluting balloon-expandable stents are frequently perfect for petrous internal carotid artery and intradural vertebral artery stenoses [23]; however, perforator basilar artery strokes with an associated atherosclerotic plaque are better treated with a Solitaire stent alone [24].

*Technical developments* for advanced treatment of ICAS are pending. The DSA-based vessel diameter measurements are frequently inaccurate, causing sizing mistakes in the balloon diameter selection. Dual antiplatelet medication is required for all possible stents and increases the risk of hemorrhagic complications. Stents, both balloon-expandable and self-expanding, with reduced surface thrombogenicity, would allow treatment under single antiplatelet medication [25].

Future trials will primarily address patients after failed medical treatment. For the time being, endovascular treatment as a first-line therapeutic option will be difficult to justify, given the good results of conservative management. A randomized trial comparing medical vs. endovascular treatment is currently beyond the horizon.
